# Overexpression of the *LcCUC2-like* gene in *Arabidopsis thaliana* alters the cotyledon morphology and increases rosette leaf number

**DOI:** 10.7717/peerj.12615

**Published:** 2022-02-02

**Authors:** Shaoying Wen, Jiayu Li, Ziyuan Hao, Lingmin Wei, Jikai Ma, Yaxian Zong, Huogen Li

**Affiliations:** Key Laboratory of Forest Genetics & Biotechnology of Ministry of Education, Co-Innovation Center for Sustainable Forestry in Southern China, Nanjing Forestry University, Nanjing, Jiangsu, China

**Keywords:** Liriodendron Chinense, Leaf development, Cotyledon, Rosette leaf, LcCUC2-like

## Abstract

**Background:**

The unique ‘mandarin jacket’ leaf shape is the most famous trait of *Liriodendron chinense* and this characteristic gives *L. chinense* aesthetic and landscaping value. However, the underlying regulatory mechanism of genes involved in the leaf development of *L. chinense* has remained unclear.

**Methods:**

Based on transcriptome data of leaves at different developmental stages from *L. chinense*, we identified differentially expression genes (DEGs) functioning in leaf development. A candidate gene named *LcCUC2-like* (*LcCUC2L*) had high similarity in sequence with *Arabidopsis thaliana CUC2*, and used for further research. We isolated the full-length *LcCUC2L* gene and its promoter from *L. chinense*. Subsequently, we analyzed the function of the *LcCUC2L* gene and its promoter activity via transformation into *A. thaliana*.

**Results:**

In this study, we found that the *LcCUC2L* and *AtCUC2* are homologous in sequence but not homologous in function. Unlike the role of *AtCUC2* in leaf serration and SAM formation, the *LcCUC2L* mainly regulates cotyledon development and rosette leaf number. Histochemical *β*-glucuronidase (GUS) staining revealed that *LcCUC2L* was expressed in the cotyledons of *A. thaliana* seedlings, indicating that the *LcCUC2L* may play a role in cotyledon development. Ectopic expression of *LcCUC2L* resulted in long, narrow cotyledons without petioles, abnormal lamina epidermis cells and defective vascular tissue in cotyledons, and these results were consistent with the* LcCUC2L* expression pattern. Further analysis showed that overexpression of *LcCUC2L* also induced numerous rosette leaves. Also, *LcCUC2L* and other related genes showed a severe response in *L. chinense* by introducing exogenous auxin stimulation, partly revealed that *LcCUC2L* affects the leaf development by regulating the auxin content.

**Conclusions:**

These results suggest that *LcCUC2L* may play a critical role in leaf development and morphogenesis in *L. chinense*, and our findings provide insight into the molecular mechanisms of leaf development in *L. chinense*.

## Introduction

The leaf, as an indispensable plant organ, functions in photosynthesis, respiration, and photoperception (Li et al., 2007). Leaf shape is one of the most important characteristics affecting plant productivity and survival. Previous studies have shown that compared with unlobed leaves, lobed leaves have larger specific leaf areas, which can enhance the competitive ability of the plant for light resources and improve photosynthetic rates (Semchenko & Zobel, 2007). Moreover, dissected leaves allow adjustment of leaf surface temperature and allow strong adaptability to stress (Vogel, 2009). In recent years, researchers have paid much attention to the regulatory mechanisms of leaf shape development (Bilsborough et al., 2011). In addition to environmental factors, many genetic factors, such as complex gene interactions, gene expression patterns, and microRNAs, and active hormonal regulation influence leaf shape development (Dkhar & Pareek, 2014). To date, many studies of leaf shape development have focused on herbaceous plants such as *A. thaliana* (Kessler & Sinha, 2004)*, Cardamine hirsuta* (Barkoulas et al., 2008), *Medicago truncatula* (Peng et al., 2011), and *Solanum lycopersicum* (Chitwood et al., 2014). However, in woody plants, the mechanisms of leaf shape development remain poorly understood.

*Liriodendron chinense* (Hemsl.) Sarg, a valuable endemic tree belonging to the magnolia family (Magnoliaceae), is distributed mainly in southern China and northern Vietnam (Fang, 1994; Wang, 1997). *L. chinense* is an attractive species for ornamental use, with a distinctive leaf shape similar to that of the traditional Chinese mandarin jacket. Normally, *L. chinense* has one deep lobe on each side of the leaf margin (Yang et al., 2014). Due to its unique leaf shape, some investigators have focused on leaf development in *L. chinense*. To date, some progress has been made in this field. For instance, a comparison of *L. chinense* leaf transcripts among various developmental stages has been performed (Ma et al., 2018). Ectopic expression of *KNOX6* from *L. chinense* in *A. thaliana* was found to result in numerous lobed leaves. It has been proposed that *LcKNOX6* might participate in leaf development in *L. chinense* (Ma et al., 2020). Also, some *LcAP2/ERFs* also seems involved in leaf morphogenesis through STC analysis in various tissues and the further anatomical assay of the leaf bud (Zong et al., 2021).

*CUC*, a member of the NAC transcription factor family with a highly conserved N-terminal domain and a variable C-terminal domain (Taoka et al., 2004), was first isolated from *A. thaliana* (Aida et al., 1997). Leaves usually originate from protuberant clusters of cells around the shoot apical meristem (SAM) (Kessler & Sinha, 2004). Experimental evidence suggests important roles of *CUC2* in SAM formation and organ boundary formation, with *cuc1 cuc2* double mutants exhibiting a single cup-shaped cotyledon and a deficiency in embryonic SAM (Aida et al., 1997). Moreover, CUC transcription factors are commonly called plant dissectors partially due to their functions in leaf margin development (Bilsborough et al., 2011). Early reports showed that the expression of the *CUC2* gene is modulated by auxin (Vernoux et al., 2000; Aida et al., 2002; Furutani et al., 2004). *PINFORMED* 1 (*PIN1*), as the main auxin transporter (Galweiler et al., 1998), is regulated by *CUC2* during serration formation (Kawamura, Horiguchi & Tsukaya, 2010). Auxin, *PIN1*, and *CUC2* have been demonstrated to compose a regulatory network involved in leaf serration formation (Bilsborough et al., 2011). Belonging to a class of plant-specific microRNAs, miR164 is a significant regulatory factor for normal plant development (Mallory et al., 2004). It has been confirmed that *CUC2* is the target gene of miR164 (Rhoades et al., 2002). miR164 negatively regulates *CUC2*, and their balance influences the depth of leaf lobation (Nikovics et al., 2006). In addition, *DEVELOPMENT-RELATED PcG TARGET IN THE APEX4* (*DPA4*) inhibits leaf serration formation by negatively regulating *CUC2* expression independent of miR164 (Engelhorn et al., 2012). Investigators have proven that *NGATHA-LIKE 1* (*NGAL1*) directly inhibits *CUC2* expression by binding to the *CUC2* promoter and thereby negatively regulates the formation of leaf margin serration (Shao et al., 2020). In addition, *cuc2 cuc3* double mutants exhibit a lack of an axillary meristem (Hibara et al., 2006), indicating that *CUC2* might be involved in axillary meristem initiation. Previous studies have suggested that *CUC2* plays important roles in adventitious shoot formation (Daimon, Takabe & Tasaka, 2003) and floral organ development (Aida et al., 1997; Baker et al., 2005; Kamiuchi et al., 2014; Gonzalez-Carranza et al., 2017). Moreover, *CUC2* plays a role in internode development, with *BpCUC2* overexpression leading to internode shortening in *Betula pendula* (Liu et al., 2019). Taken together, these observations suggest that *CUC2* participates in both growth and development in plants.

In this study, *LcCUC2L* and its promoter were isolated from *L. chinense* and characterized by conducting bioinformatics analysis. Assessments of the functions of *LcCUC2L* in transgenic *A. thaliana* plants were performed using overexpression assays. As a result, GUS staining revealed that *LcCUC2L* was expressed in the cotyledons of *A. thaliana* seedlings, which indicated that *LcCUC2L* may play a role in cotyledon development. Intriguingly, almost all the transgenic *A. thaliana* lines displaying abnormal cotyledons and an increased rosette leaf number. Further SEM observation and venation pattern analysis showed that overexpression of *LcCUC2L* induced abnormal lamina epidermis cells and defective vascular tissue of cotyledons. Hormone determination and RT-qPCR results indicated that *LcCUC2L* affects leaf development by regulating the auxin content and the expression of genes involved in auxin synthesis, transport, and leaf shape development in *A. thaliana*. Our results will help to reveal the molecular mechanisms of leaf development in *L. chinense*.

## Materials & Methods

### Plant materials, growth conditions, and treatments

Samples of leaves at different developmental stages (Tu et al., 2019) were harvested from an adult *L. chinense* tree in Xiashu, Jurong County, Jiangsu Province, China (119°13′20^′^E, 32°7′8^′^N) (Yang et al., 2014). Plant materials were maintained at −80 °C prior to analysis.

Wild-type (WT) *A. thaliana* (Columbia-0), transgenic *A*. *thaliana, Nicotiana benthamiana* (*Ben*) plants, and *L. chinense* seedlings were cultivated at 23 °C under a long-day photoperiod (16 h light, 8 h dark) in a growth chamber (70% relative humidity).

For the IAA treatment, *L. chinense* seedlings grown in the incubator for 5 months were sprayed with 200 µM IAA on leaves and then sampled at 48 h. Water was used as the control. The samples were placed in an ultra-low-temperature freezer for further analysis.

### DNA, RNA extraction, and RT-qPCR

A DNAsecure Plant Kit (Tiangen) was used for DNA isolation from *L. chinense*. RNA was extracted from *L. chinense* and *A. thaliana* leaves using the RNAprep Pure Plant Kit (Tiangen). Subsequently, first-strand cDNA was synthesized using PrimeScript™ RT Master Mix (TaKaRa) in accordance with the manufacturer’s protocol. The *LcCUC2L* transcript levels in leaves of different developmental stages (Tu et al., 2019) were investigated by RT-qPCR. Moreover, we also detected *LcCUC2L* expression levels under IAA treatment. *L. chinense* Actin 97 was used as the internal control. In addition, the transcription levels of *LcCUC2L* and some other genes in WT and transgenic lines were examined using specific primers (Table S1). *A. thaliana* Actin 2 was used as the internal quantitative control. RT-qPCR was carried out using the SYBR Premix EX Taq kit (TaKaRa). We applied the 2^−ΔΔ*C*^_T_ method to analyze the data from relative quantification (Livak & Schmittgen, 2001).

### Cloning and bioinformatics analysis

Based on the known transcriptome database (Ma et al., 2018), the *CUC2* gene was identified in *L. chinense*. The full-length *LcCUC2L* gene was obtained using RT-PCR and RACE. The *LcCUC2L* gene was assembled with the middle region, 5^′^ sequence and 3^′^ sequence. ORF Finder was used for prediction of the open reading frame (ORF) and protein sequence. The structure of the *LcCUC2L* gene was analyzed by the online software GSDS (http://gsds.gao-lab.org/). Analysis of the physicochemical characteristics of the proteins was conducted online at the following website: (https://web.expasy.org/protparam/). PredictProtein was used to forecast protein secondary structure. Subcellular localization was predicted by applying WoLF PSORT. Protein conserved domain analysis of LcCUC2L was performed with NCBI (https://www.ncbi.nlm.nih.gov/cdd/?term). The amino acid sequences of the *CUC2* gene in other plant species were acquired from GenBank (http://www.ncbi.nlm.nih.gov/genbank). Multiple sequence alignment of LcCUC2L and its homologous genes was performed with ClustalW (https://www.genome.jp/tools-bin/clustalw). The results were uploaded to ESPript 3.0 (http://espript.ibcp.fr/ESPript/cgi-bin/ESPript.cgi). A phylogenetic tree was constructed using MEGA 7.

After consulting the published *L. chinense* genome data (Chen et al., 2019), approximately 2 kb of genomic DNA upstream of the start codon of *LcCUC2L* was cloned. The PlantCARE (http://bioinformatics.psb.ugent.be/webtools/plantcare/html) was used to predict the cis-acting elements of *ProLcCUC2L*.

### Subcellular localization of *LcCUC2L*

The whole coding sequence (CDS) of *LcCUC2L* without a stop codon was ligated to a linearized *pBI121-eGFP* vector. The recombinant vector was transformed into *Agrobacterium tumefaciens* GV3101 (ShiFeng). And the 35S::*eGFP* was used as the control. Positive clones and the *P19* vector were cultured in Luria-Bertani (LB) medium at 28 °C. Next, the cells were collected and suspended in buffer containing 10 mM MgCl2 and 150 *μ*M acetosyringone (AS). Two bacterial suspensions were mixed and injected into tobacco leaves (Sparkes et al., 2006). After 48 h of injection, the tobacco leaves were stained with the nuclear dye 4^′^, 6-diamidino-2-phenylindole (DAPI) to visualize the nucleus. Subsequently, the fluorescent signals in the epidermis cells of tobacco leaves were examined with a confocal laser scanning microscope (LSM 710, Zeiss, Germany).

### Plasmid construction and genetic transformation

The full-length ORF of *LcCUC2L* was cloned into the linearized overexpression vector *pBI121*. Similarly, the sequence of *ProLcCUC2L* was inserted at the *HindIII/NcoI* site of the *pCAMBIA1301* vector by substituting the CaMV35S promoter, yielding the construct *ProLcCUC2L::GUS*. The *Agrobacterium*-mediated floral dip method was used for the genetic transformation of *A. thaliana* (Clough & Bent, 1998). Transgenic *A. thaliana* seeds were screened on $ \frac{1}{2} $ MS medium containing antibiotics. Using leaf genomic DNA as templates, T1-positive transgenic plants were identified by PCR using specific primers. We acquired T3 transgenic plants through continuous screening for three generations.

### Venation pattern analysis and scanning election microscopy

To compare cotyledon vein patterns of transgenic and WT *A. thaliana* plants, the cotyledons were bleached with 75% (v/v) ethanol and observed using stereomicroscopy. For cotyledon epidermal cell analysis, the cotyledons of transgenic and WT *A. thaliana* plants were fixed in FAA fixative solution (38% formalin, 50% ethanol, and glacial acetic acid). After critical point drying (CPD), the samples were coated with an Edwards E-1010 ion sputter golden coater and examined with a scanning electron microscope (FEI Quanta 200 FEG MKII).

### Auxin content measurement

Basal rosette leaves samples of 0.6 g were harvested from 3-week-old WT and *LcCUC2L* transgenic *A. thaliana* plants and immediately frozen in liquid nitrogen. Determination of indole-3-acetic acid (IAA) in *A. thaliana* leaves was performed by ELISA (Huding, Shanghai, China). Three replicates were used for each sample.

### Histochemical staining to detect GUS activity

The *β*-Galactosidase Reporter Gene Staining Kit (Leagene) was used for GUS histochemical staining in *ProLcCUC2L* transgenic and WT *A. thaliana* plants. In this study, seven-day-old seedlings and fourteen-day-old seedlings were processed. Moreover, leaves at different stages of development (10, 20 and 30 days of age) from plants planted in soil were subjected to GUS staining. All materials were treated with GUS staining solution and incubated overnight at 37 °C, followed by bleaching with 75% (v/v) ethanol.

### Statistical analysis

Both the control and the experimental lines had three repetitions. The data are expressed as the mean ± standard deviation (SD). Statistical significance was determined with Student’s *t*-test. *P* < 0.05 was regarded as significant (*), and *P* ≤ 0.01 was considered highly significant (**).

## Results

### Cloning and sequence analysis of LcCUC2L and ProLcCUC2L

**Figure 1:**
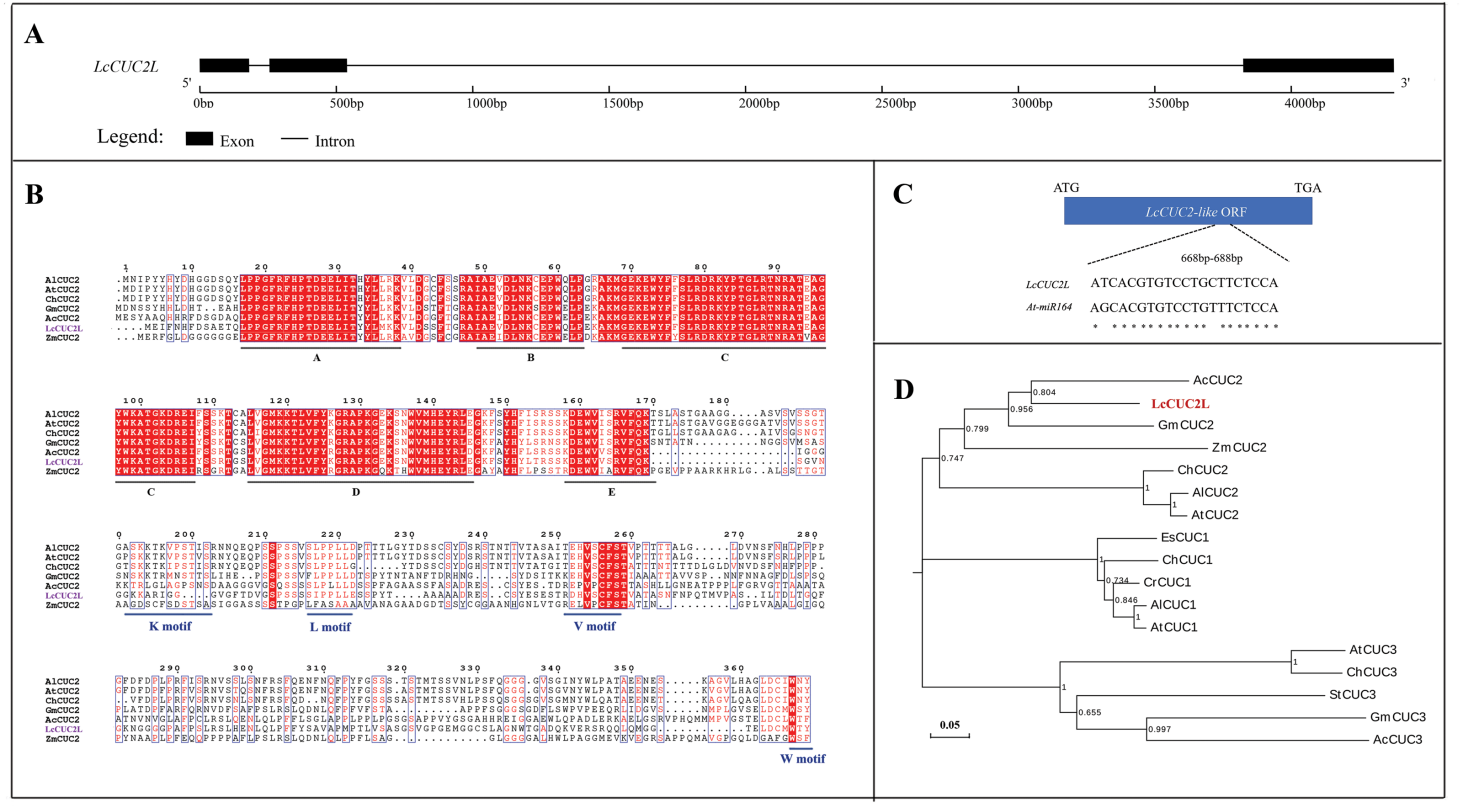
Sequence analysis of *LcCUC2L*. (A) *LcCUC2L* gene structure diagram. (B) Homology analysis of the LcCUC2L protein. Amino acid sequence alignment of LcCUC2L and CUC2 proteins in other plants. AlCUC2: *Arabidopsis lyrata subsp. lyrata* (XP_002866009.1), AtCUC2: *Arabidopsis thaliana* (OAO90779.1), ChCUC2: *Cardamine hirsuta* (ACL14370.1), GmCUC2: *Glycine max* (XP_003541838.1), AcCUC2: *Ananas comosus* (OAY67566.1), and ZmCUC2: *Zea mays* (PWZ16696.1). (C) Nucleotide sequence alignment of the *LcCUC2L* and *AtCUC2* that targeted by miR164. (D) Phylogenetic analysis of LcCUC2L and CUC proteins from other plants. EsCUC1: *Eutrema salsugineum* (XP_006407005.1), ChCUC1: *Cardamine hirsute* (ACL14369.1), CrCUC1: *Capsella rubella* (XP_006296616.1), AlCUC1: *Arabidopsis lyrata subsp. lyrata* (XP_002882916.1), AtCUC1: *Arabidopsis thaliana* (BAB20598.1), AlCUC2: *Arabidopsis lyrata subsp. lyrata* (XP_002866009.1), AtCUC2: *Arabidopsis thaliana* (OAO90779.1), ChCUC2: *Cardamine hirsuta* (ACL14370.1), GmCUC2: *Glycine max* (XP_003541838.1), AcCUC2: *Ananas comosus* (OAY67566.1), ZmCUC2: *Zea mays* (PWZ16696.1), AtCUC3: *Arabidopsis thaliana* (AAP82630.1), GmCUC3: *Glycine max* (XP_003523523.1), StCUC3: *Solanum tuberosum* (NP_001275002.1), ChCUC3: *Cardamine hirsuta* (ACL14365.1), and AcCUC3: *Aquilegia coerulea* (ACL14364.1).

The full length of the *LcCUC2L* gDNA sequence is 4,374 bp and consists of three exons and two introns (Fig. 1A). *LcCUC2L* (accession number: lcCUC2MW629054) contains a 1,017 bp ORF (Data S1), which encodes 338 amino acids and has a predicted molecular weight of 37.32 kDa and a theoretical PI of 8.45. The instability index was 44.46, indicating that the protein is unstable. The secondary structure of *LcCUC2L* was predicted to consist of helix (3.25%), loop (84.02%) and strand (12.72%) structures. Subcellular localization prediction showed that the LcCUC2L protein was mainly distributed in the cell nucleus. The amino acid sequences of the LcCUC2L protein in *L. chinense* and other plants were analyzed *via* multiple sequence alignment. Furthermore, the amino acid sequence of LcCUC2L was compared with the sequences of other CUC2 proteins in *Arabidopsis lyrata* subsp. *lyrata*, *A. thaliana*, *Ananas comosus*, *Cardamine hirsute*, *Glycine max*, and *Zea mays*. Similar to the N-terminus of other CUC2 proteins, the N-terminus of the LcCUC2L protein contains a conserved NAC domain, with five conserved subdomains (A, B, C, D, E), indicating that these proteins belong to the CUC subfamily of the NAC family. In addition, CUC genes harbor a variable CTD domain. The K motif (PSKKTKVPSTIS), V motif (EHVSCFS), L motif (SLPPL) and W-motif (WNY) can be observed in CUC2. In contrast, CUC1 does not have a K motif and there is only an L motif in CUC3 (Larsson et al., 2012). LcCUC2L harbors a variable CTD domain, and the K motif, L motif, V motif and W motif were observed in the LcCUC2L protein (Fig. 1B). The V motif of CUC1 and CUC2 has been identified as a miR164 recognition site (Rhoades et al., 2002). As shown in Fig. 1C, which indicated the *LcCUC2L* might be recognized by miR164. Moreover, phylogenetic tree analysis confirmed that CUC proteins could be classified into three clads: CUC1, CUC2, and CUC3. The LcCUC2L protein is most closely related to the *Ananas comosus* CUC2 protein (Fig. 1D). In conclusion, these results show that the sequence is highly homologous to CUC2, so we named it *LcCUC2L*.

To explore the function of *LcCUC2L*, a 2,028 bp (Data S2) sequence upstream of the translational initiation site (ATG) of the *LcCUC2L* gene, which contains the promoter sequence of *LcCUC2L*, was amplified using the DNA template of *L. chinense*. PlantCARE software was used to analyze the *cis*-acting elements of *ProLcCUC2L*. As shown in Table 1, some cis-acting elements are involved in light regulation, such as AE-box, ATCT-motif, Box4, G-Box, G-box, I-box, TCCC-motif, and chs-CMA1a. LTR and WUN motifs associated with low-temperature responsiveness and wound responsiveness, respectively, exist in the sequence. In addition, some hormone-related elements were identified, including the abscisic acid responsive regulatory motif (ABRE), auxin-responsive element (TGA-element) and MeJA-responsiveness elements (CGTCA-motif and TGACG-motif). The predicted results revealed that the core promoter elements of *ProLcCUC2L* contained TATA-box and CAAT-box.

**Table 1:** Details of cis-acting elements of the *ProLcCUC2L* in *L. chinense*.

Name	Number	Sequence	Function
ABRE	3	CACGTG/ ACGTG	cis-acting element involved in the abscisic acid responsiveness
AE-box	1	AGAAACAA	part of a module for light response
ARE	2	AAACCA	cis-acting regulatory element essential for the anaerobic induction
ATCT-motif	1	AATCTAATCC	part of a conserved DNA module involved in light responsiveness
Box 4	1	ATTAAT	part of a conserved DNA module involved in light responsiveness
CAAT-box	27	CAAT/CAAAT	common cis-acting element in promoter and enhancer regions
CGTCA-motif	3	CGTCA	cis-acting regulatory element involved in the MeJA-responsiveness
G-Box	2	CACGTGAAA/ CACGTG	cis-acting regulatory element involved in light responsiveness
G-box	2	CACGTG/CACGTC	cis-acting regulatory element involved in light responsiveness
I-box	1	atGATAAGGTC	part of a light responsive element
LTR	1	CCGAAA	cis-acting element involved in low- temperature responsiveness
TATA-box	8	TATAA/TATA/ ccTATAAAaa/TATACA	core promoter element around -30 of transcription start
TCCC-motif	1	TCTCCCT	part of a light responsive element
TGA-element	1	AACGAC	auxin-responsive element
TGACG-motif	3	TGACG	cis-acting regulatory element involved in the MeJA-responsiveness
chs-CMA1a	1	TTACTTAA	part of a light responsive element
WUN-motif	1	AAATTTCCT	wound-responsive element

### Expression pattern analysis of *LcCUC2L*

**Figure 2:**
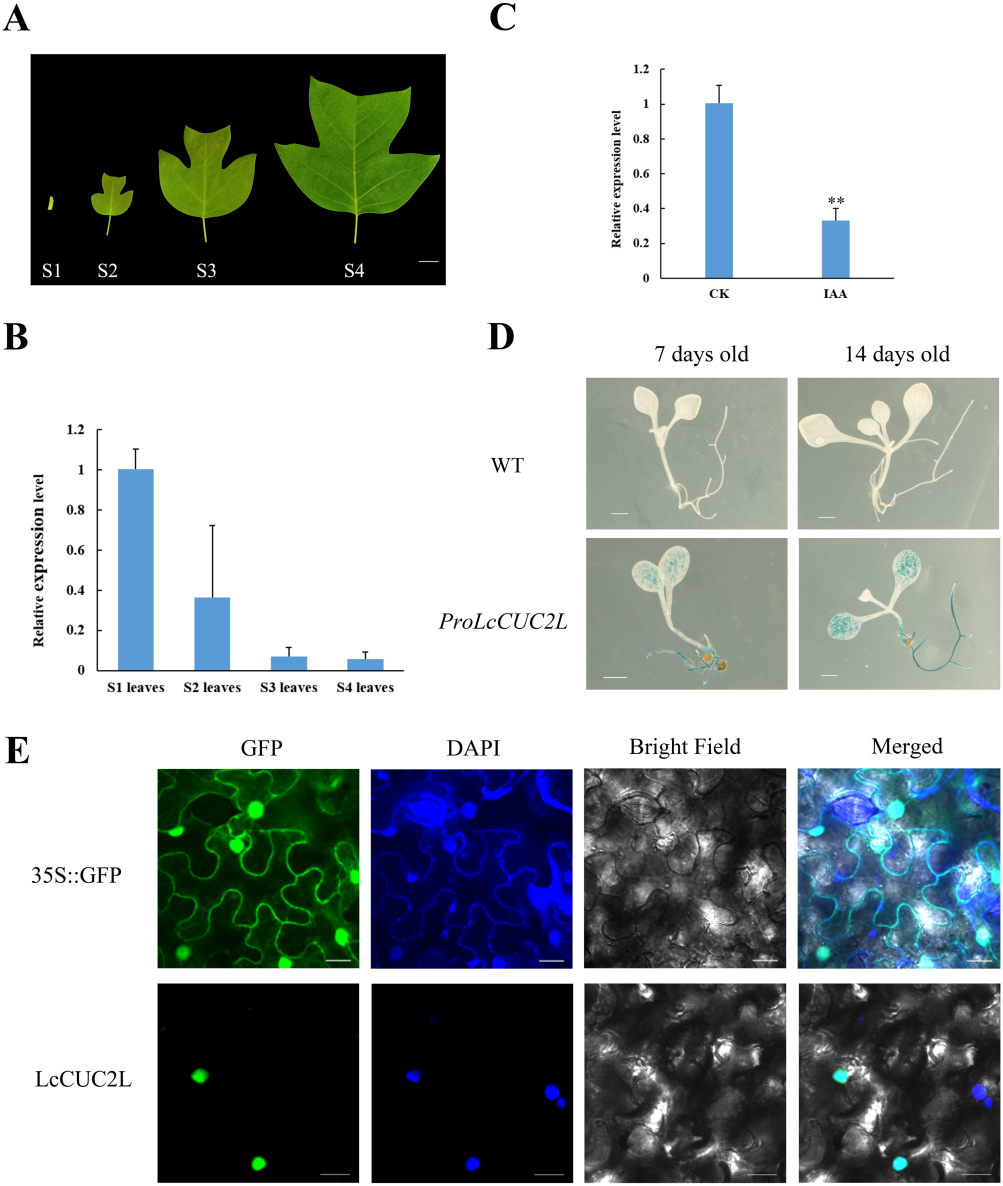
Expression pattern analysis of *LcCUC2L*. (A) Leaves of four different developmental stages in *L. chinense*. S1 leaves: leaf bud; S2 leaves: newly expanded leaves; S3 leaves: larger leaves; S4 leaves: mature leaves, bars = two cm. (B) Transcript levels of *LcCUC2L* in leaves of different developmental stages in *L. chinense*. (C) Expression level of *LcCUC2L* under 200 µM IAA. (D) Histochemical analysis of GUS in transformed *A. thaliana* leaves, bars = 0.1 cm. (E) Subcellular localization of LcCUC2L protein, bars = 2,000 nm. Note: In B and C data are shown as the mean ± SD, B and C data are based on three repetitive experiments. **P < 0.01 and *P < 0.05, Student’s *t*-test.

The transcript levels of *LcCUC2L* were determined by RT-qPCR in leaves of different developmental stages (Fig. 2A). The highest expression levels of *LcCUC2L* were observed in the leaf bud, and the lowest abundance of *LcCUC2L* transcripts were in mature leaves (Fig. 2B, Table S2). The results indicated that *LcCUC2L* may play an important role in leaf bud development.

Based on cis-element sequence analysis of the *LcCUC2L* promoter, we predicted that *LcCUC2L* might be associated with auxin. To confirm the effect of auxin on *LcCUC2L* expression, we sprayed 200*μ*M IAA on the leaves of *L. chinense*. The application of IAA significantly inhibited the expression of *LcCUC2L* (Fig. 2C, Table S3), which suggested that IAA represses *LcCUC2L* expression.

To further assess the expression pattern of *LcCUC2L*, *ProLcCUC2L* was fused to the GUS reporter and subsequently transferred into *A. thaliana* (hereafter *ProLcCUC2L*). After screening in media containing hygromycin and DNA detection, we obtained 8 transgenic lines (Fig. S1). Histochemical GUS staining was applied to detect the expression levels of the GUS gene in transgenic *A. thaliana* plants, which displayed inducible activity of *ProLcCUC2L*. In the 7-day-old and 14-day-old transgenic seedlings, GUS staining was mainly detected in the cotyledon lamina and roots (Fig. 2D). In contrast, low GUS activity was observed in the cotyledon petiole and hypocotyl of the transgenic seedlings. GUS activity was not detected in the true leaves of 14-day-old transgenic seedlings. These results indicated that *LcCUC2L* may have participated in cotyledon development in the transgenic *A. thaliana* seedlings.

Confocal images with DAPI nuclear staining (blue) were taken 48 h after transfection, showing GFP (green) expression that indicates the subcellular localization of LcCUC2L. Figure 2E shows that the green fluorescence of the control *35S::GFP* was distributed throughout the cells. In contrast, the green fluorescence of the *35S::LcCUC2L-eGFP* fusion protein was observed in the nucleus, suggesting that LcCUC2L localized to the nucleus. This result was fully consistent with the subcellular localization result of the AtCUC2 protein in *A. thaliana* (Taoka et al., 2004).

### Overexpression of *LcCUC2L* regulates leaf development in *A. thaliana*

**Figure 3:**
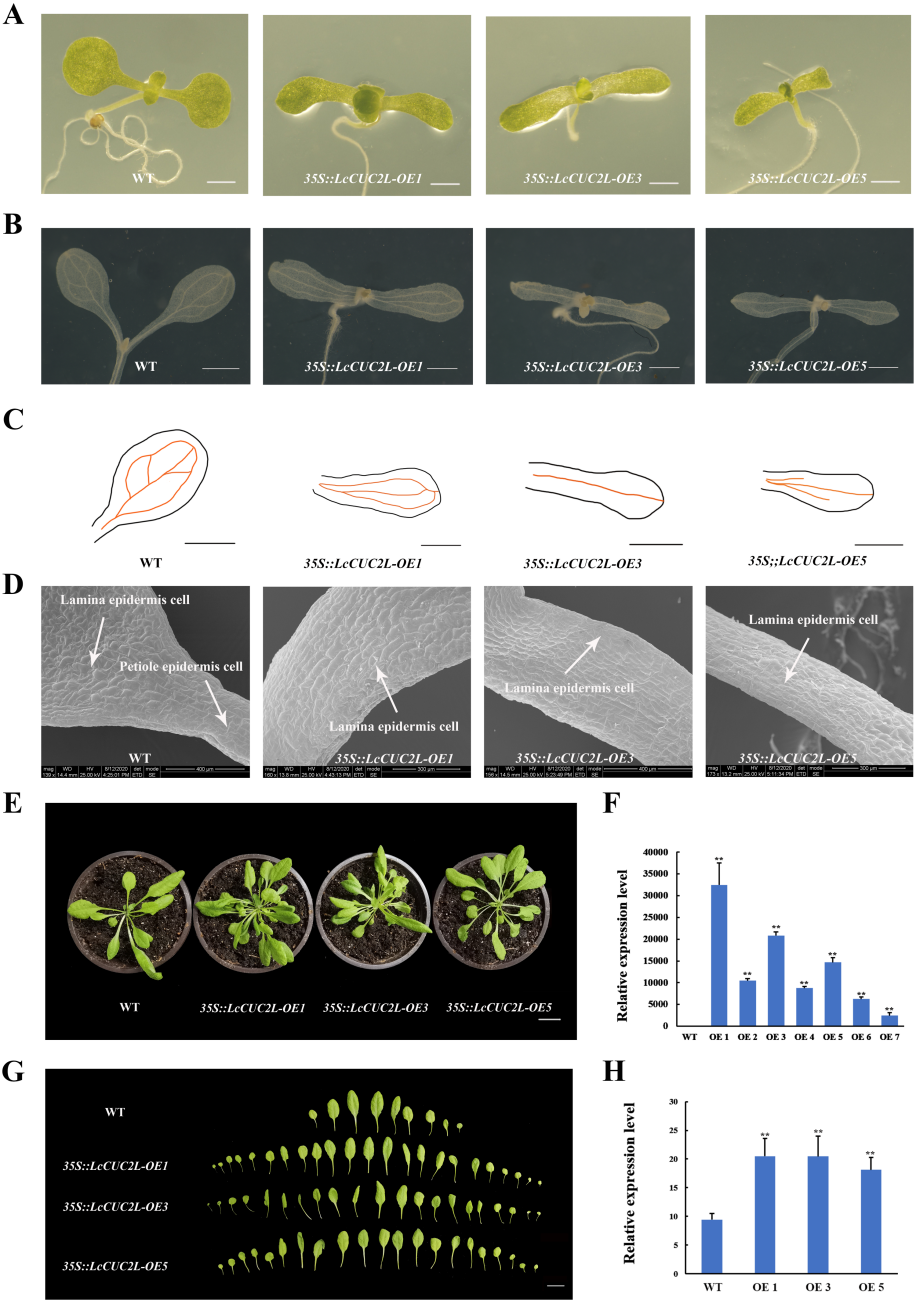
The phenotypes of the *LcCUC2L* overexpression transgenic lines and WT *A. thaliana* plants. (A) Seven-day-old cotyledons of WT and *35S::LcCUC2L* plants, bars = 0. 1 cm. (B) Venation patterns of WT and *35S::LcCUC2L* plants, bars = 0.1 cm. (C) Illustrations of the vascular patterns of 7-day-old cotyledons of WT and *35S::LcCUC2L* plants. (D) SEM images of cotyledons of WT and *35S::LcCUC2L* plants. (E) 30-day-old WT and *35S::LcCUC2L A. thaliana*, bars = 2 cm. (F) qRT-PCR analysis of *LcCUC2L* expression in WT and transgenic plants. Values are means ± SD (*n* = 3). Data are based on three repetitive experiments. **P < 0.01 and *P < 0.05, Student’s *t*-test. (G) The rosette leaves of WT and *35S::LcCUC2L A. thaliana*, bars = 2 cm. (H) The number of rosette leaves in WT and *35S::LcCUC2L A. thaliana*. Values are means ± SD (*n* = 18). **P < 0.01 and *P < 0.05, Student’s *t*-test.

To investigate the functions of *LcCUC2L*, we obtained transgenic *A. thaliana* plants overexpressing *LcCUC2L* under the control of the *CaMV 35S* promoter. After screening on selective medium, seven positive transgenic plants were acquired. Subsequently, three high expression lines with the same phenotypes (OE 1, OE 3, and OE 5) were selected for phenotype analysis (hereafter *35S::LcCUC2L-OE1*, *35S::LcCUC2L-OE3*, and *35S::LcCUC2L-OE5*) (Fig. 3F, Table S4). We found that true leaves began to emerge on or near the 7th day, as shown in Fig. 3A. The results revealed significant variation in the cotyledon phenotypes between WT and *35S::LcCUC2L* plants. In contrast to WT cotyledons, the cotyledons of the *35S::LcCUC2L* plants appeared as long strips and without petioles, and they had narrower blades compared to the WT cotyledons. Moreover, we compared vascular development between the WT and *35S::LcCUC2L* cotyledons. Two kinds of veins exist in WT cotyledons: primary veins and subprime veins. Several subprime veins branch from the midvein and then unite to form areoles separated by veins (Figs. 3B, 3C) (Sieburth, 1999). In contrast, *35S::LcCUC2L* plant cotyledons displayed an incomplete vascular pattern and had only a single central strand (Figs. 3B, 3C). In conclusion, the *35S::LcCUC2L* cotyledons exhibited significant deficiencies in vascular development. To examine the cotyledon cell changes in the transgenic plants, we imaged the epidermal cells of *35S::LcCUC2L* and WT with scanning election microscopy (SEM). Scanning election microscopy observations revealed that in WT cotyledons, the epidermal cells were organized in a specific pavement-like pattern (Fig. 3D) (Koyama et al., 2007). Remarkably, the shape and arrangement of the epidermal cells in the *35S::LcCUC2L* cotyledons were similar to those of the WT petiole, with both being arranged regularly and exhibiting a rectangular shape. Taken together, the findings revealed large variations in cotyledon morphology between the *35S::LcCUC2L* and WT *A. thaliana* plants, demonstrating that *LcCUC2L* affects cotyledon development in *A. thaliana*.

Ten days later, we transplanted seedlings of the homozygous transgenic lines and WT *A. thaliana* plants into nutrient soil. We observed that the rosette leaves of the 30-day-old *35S::LcCUC2L* plants grew in clusters and were small and numerous (Figs. 3E, 3G). The *35S::LcCUC2L-OE1* and *35S::LcCUC2L-OE3* had 20 rosette leaves on average and the *35S::LcCUC2L-OE5* had 18 rosette leaves on average (Table S5), which was considerably more than the number in WT (Fig. 3H). These results indicated that *LcCUC2L* influences leaf development in *A. thaliana*.

### *LcCUC2L* regulates leaf development by upregulating the expression of some genes related to auxin and leaf shape development

**Figure 4:**
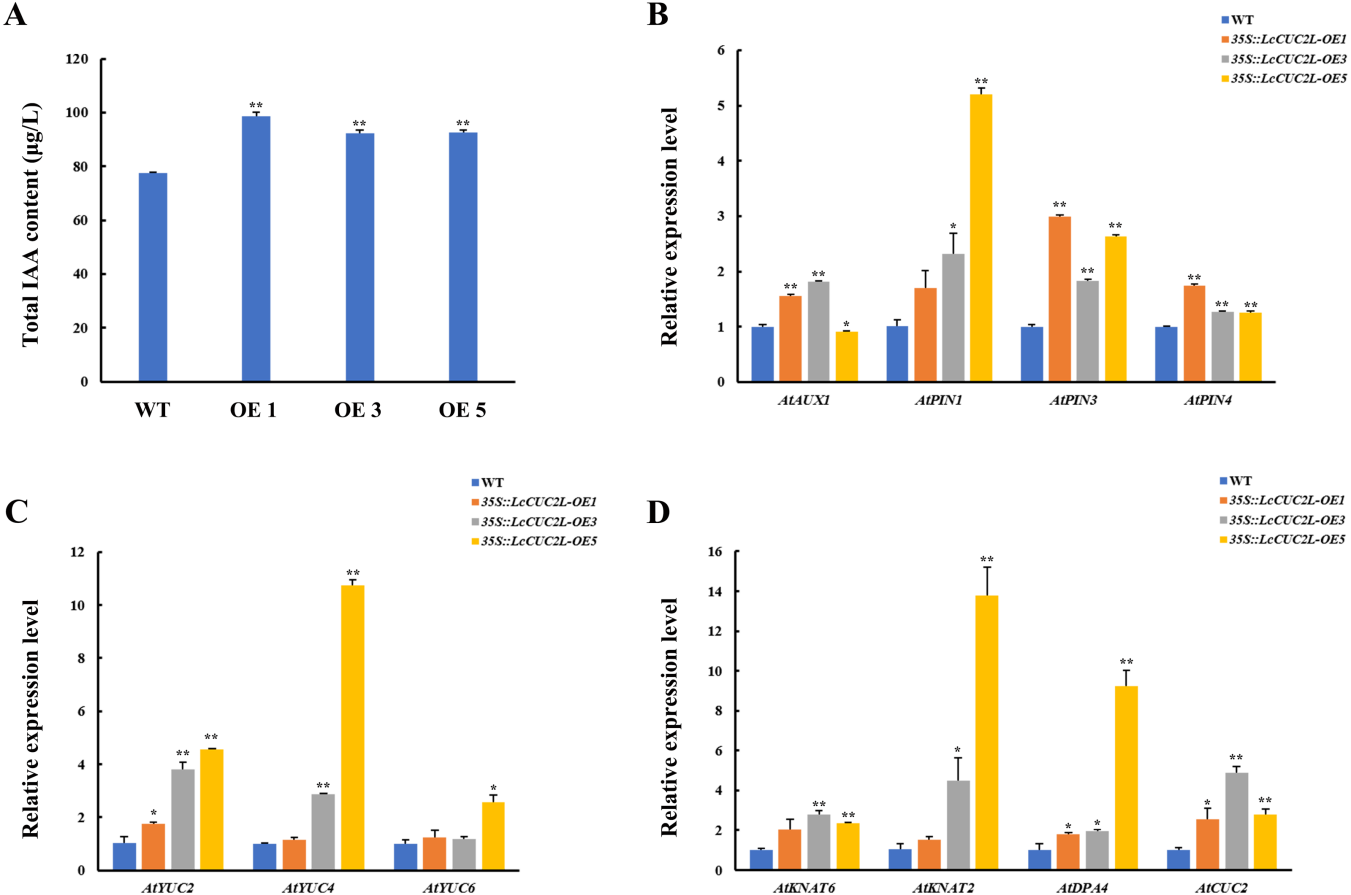
*LcCUC2L* promoted auxin accumulation in leaves and upregulated the expression of some genes in transgenic *A. thaliana* plants (*35S::LcCUC2L*). (A) Total IAA content in the rosette leaves of three-week-old seedlings of WT and *35S::LcCUC2L A.* thaliana plants. (B) Transcript levels of auxin biosynthetic genes (*AtYUC2*, *AtYUC4*, and *AtYUC6*) in WT and *35S::LcCUC2L A. thaliana* plants. (C) Transcript levels of auxin influx carriers (*AtAUX1*) and efflux carriers (*AtPIN1*, *AtPIN3*, and *AtPIN4*) in WT and *35S::LcCUC2L A. thaliana* plants. (D) Transcript levels of some genes involved in the leaf shape development (*AtKNAT2*, *AtKNAT6*, *AtDPA4*, and *AtCUC2*) in WT and *35S::LcCUC2L A. thaliana* plants. Note: In A, B, C, and D data are shown as the mean ± SD, based on three repetitive experiments. **P < 0.01 and *P < 0.05, Student’s *t*-test.

Many recent investigations have indicated that auxin plays a vital role in leaf development (Xiong & Jiao, 2019). The *35S::LcCUC2L* plants amassed more IAA than the WT *A. thaliana* plants (Fig. 4A, Table S6). To explore whether *LcCUC2L* regulates leaf development by affecting auxin signaling, the expression levels of auxin biosynthesis genes and auxin transport genes were examined (Table S7). The transcript levels of the auxin biosynthetic genes *YUCCA* (*AtYUC2* and *AtYUC4*) (Fig. 4B), an auxin influx carrier (*AtAUX1*), and efflux carriers (*AtPIN1*, *AtPIN3*, and *AtPIN4*) (Fig. 4C) were increased in *LcCUC2L*-expressing plants compared to WT *A. thaliana* plants. However, *LcCUC2L* overexpression had little effect on the expression level of *AtYUC6*. The results imply that the effects of *LcCUC2L* in leaf development might be enhanced by changes in auxin biosynthesis and polar transport. Additionally, we analyzed some genes related to leaf shape development, *i.e., KNAT1*, *KNAT2*, *KNAT6* (for *KNOTTED*-like from *A. thaliana*), *SHOOOTMERISTEMLESS* (*STM*), and *DPA4*. We found that *35S::LcCUC2L* plants presented much higher transcript levels of *KNAT6*, *KNAT2*, *DPA4*, and *AtCUC2* than WT *A. thaliana* plants (Fig. 4D). The results indicate that the overexpression of *LcCUC2L* led to the upregulation of some genes related to leaf shape development and thus affected leaf development in *A. thaliana*.

## Discussion

Leaf shape is an important plants traits. Early studies of leaf shape development have focused on model plants, but there is still limited in woody plants. *L. chinense* has a distinctive leaf shape (Fig. 2A), which provides a wealth of reference information to uncover the molecular mechanisms of leaf shape development. Based on a comparison of *L. chinense* leaf transcripts among different developmental stages, the *CUC2* gene was identified as a candidate gene related to leaf development (Ma et al., 2018). Subsequently, we identified the full-length sequences of *LcCUC2L* and its promoter isolated from *L. chinense* and analyzed their functions *via* overexpression analysis.

In this study, we discovered that overexpression of *LcCUC2L* strongly affected cotyledon development in transgenic *A. thaliana*, resulting in long, narrow cotyledons without petioles (Fig. 3A) but with abnormal epidermal cells (Fig. 3D). In the early stages of leaf organogenesis, the proximal–distal, medial–lateral and adaxial–abaxial axes are established (Tsukaya, 2006; Kidner & Timmermans, 2007). The proximal–distal axis domains of WT *A. thaliana* leaves are composed of the lamina and the petiole (Jover-Gil et al., 2012). In the present study, abnormal epidermal cells were present in the cotyledons of the *35S::LcCUC2L* plants, with those in the lamina resembling the epidermal cells of the wild-type petiole (Fig. 3D). Previous studies have reported blade growth on the petiole and a diminished proximal domain in the *bop1 bop2* double mutant (Ha et al., 2007). In addition, the *as2* mutation causes the petiole to curl upward and to sometimes produce leaflets (Iwakawa et al., 2007). These phenotypes are associated with the ectopic expression of meristem class I homeobox *KNOX* genes, *i.e., KNAT2*, *KNAT6*, *STM* and *BP* (Ha et al., 2003; Iwakawa et al., 2007; Ikezaki et al., 2010; Jun, Ha & Fletcher, 2010). To our surprise, overexpression of *LcCUC2L* in *A. thaliana* promoted the expression of *KNAT2* and *KNAT6* (Fig. 4D). Further analyses suggested that the ectopic expression of *LcCUC2L* regulated the proximal–distal axis by enhancing the expression of *KNAT2* and *KNAT6*; thus, the cotyledons of *35S::LcCUC2L A. thaliana* tended to develop as long, narrow cotyledons without petioles and exhibited abnormal epidermal cells.

Furthermore, cotyledon vein development in *35S::LcCUC2L* plants was defective (Figs. 3B, 3C). It is widely known that auxin plays an indispensable role in vascular development (Aloni, 1995). Mutations in some genes related to the auxin signaling pathway (*AUXIN RESPONSE FACTOR 5* (*ARF5*), *AUXIN RESISTANT 6* (*AXR6*) and *IAA12*) contribute to incomplete vascular development (Przemeck et al., 1996; Hobbie et al., 2000; Hamann et al., 2002). Moreover, evidence suggests that polar auxin transport plays a significant role in determining leaf vascular patterns. Studies have shown that the expression of *CUC* genes is regulated by *PIN1* (Aida et al., 2002) and that blocking PIN-mediated polar auxin transport (PAT) results in cotyledon fusion (Liu, Xu & Chua, 1993). It has been proposed a feedback regulation network of leaf margin development among auxin, *PIN1*, and *CUC2* (Maugarny et al., 2016). The auxin content and *PIN1* transcript level of *35S::LcCUC2L* plants were higher than those of WT *A. thaliana* plants in this study (Figs. 4A, 4C). Bioinformatic analysis revealed that the auxin-responsive element is present in *ProLcCUC2L* (Table 1). The authors identified three upstream transcription factors that bind to the auxin response element (CACATG) of the *BpCUC2* promoter (Liu et al., 2018). Our results revealed that the expression of *LcCUC2L* markedly decreased under IAA treatment (Fig. 2C). These findings imply that *LcCUC2L* may be related to the metabolism of auxin. Based on the above results, we speculate that *LcCUC2L* elevated auxin content by upregulating local auxin biosynthesis and polar transport, which affected cotyledon development. Moreover, GUS activity was detected in the cotyledons of transgenic *A. thaliana* seedlings (Fig. 2D). This finding is in line with the fact that *LcCUC2L* regulates cotyledon development in transgenic *A. thaliana* plants.

Ectopic expression of *LcCUC2L* produced numerous rosette leaves (Figs. 3G, 3H). Leaves are lateral organs that develop continuously at the flanks of the SAM in flowering plants (Kessler & Sinha, 2004). The cotyledons of *cuc1 cuc2* double mutant seedlings are fused into a single cup-shaped cotyledon without a SAM (Aida et al., 1997). In addition, the *KNOX* gene is required to maintain the development and function of the SAM (Scofield, Dewitte & Murray, 2014). Overexpression of *LcCUC2L* led to upregulated expression of *KNAT2* and *KNAT6* (Fig. 4D), indicating that *LcCUC2L* may regulate SAM development by affecting the expression of *KNAT2* and *KNAT6*. Moreover, *LcCUC2L* was prominently expressed in the leaf buds (Fig. 2A), which is in accordance with *BpCUC2* having the highest expression level in buds (Liu et al., 2018), suggesting that *LcCUC2L* may function in the early stage of leaf development. SAM and leaf tissue can be observed in the leaf buds of *L. chinense* (Ma et al., 2018). We assumed that the development of the SAM was influenced by the overexpression of *LcCUC2L*, which increased the number of rosette leaf. These findings call for further research to verify this possibility.

Research has demonstrated that the *CUC1* and *CUC2* genes descended from a common ancestor that has undergone two duplications and sequence loss events in the evolutionary process (Bowers et al., 2003). Previous data have shown no obvious divergence between *CUC2* and the ancestral gene, while *CUC1* has diverged from the ancestral gene, resulting in a different function (Hasson et al., 2011). For example, both *CUC1* and *CUC2* participate in primordium development, while *CUC2* and *CUC3* were proven to be involved in leaf margin development (Nikovics et al., 2006; Hasson et al., 2011). Moreover, the phylogenetic tree showed that *LcCUC2L* and *AtCUC1* were not clustered in one branch (Fig. 1D). We found that LcCUC2L had the nearest evolutionary distance to AcCUC2 (Fig. 1D) and the amino acid sequence similarity of LcCUC2L and AtCUC2 was 52.36%. These results indicated that there may be functional differentiation between *LcCUC2L*, *AtCUC2* and *AtCUC1*.

Studies in A. thaliana have suggested that *cuc1 cuc2* double mutants showed cotyledon fusion and defective SAMs, revealing a key role for *CUC* genes in cotyledon development and SAM formation (Aida et al., 1997). However, our study showed that overexpression of *LcCUC2L* strongly conferred long, narrow cotyledons without petioles (Fig. 3A) and abnormal epidermal cell (Fig. 3D) phenotypes. Moreover, *LcCUC2L* showed strong expression in cotyledons (Fig. 2D), which further confirmed that *LcCUC2L* might play a role in regulating cotyledon development. In *A. thaliana* seedlings, *AtCUC2* was expressed in the boundaries between the cotyledons, the first true leaves, and the SAM. This finding is in line with *CUC2* being a boundary-specific gene (Larsson et al., 2012; Takada et al., 2001). During leaf development, GUS activity tended to become weaker in the sinus of the leaves, which indicated that local inhibition of the *CUC2* gene may cause serration (Nikovics et al., 2006). *A. thaliana* has serrated leaves, and a smooth leaf margin phenotype was observed in the *cuc2* mutant, suggesting that *CUC2* participates in leaf serration in *A. thaliana* (Hasson et al., 2011). Accordingly, it can be inferred that the expression pattern of the *AtCUC2* gene is closely related to its function. However, the *LcCUC2L* gene was highly expressed in the cotyledons and affected the cotyledon development. Moreover, the *ProLcCUC2-GUS* line showed strong expression in the roots as well as cotyledons (Fig. 2D). This phenomenon interested us, and the role of *LcCUC2L* in root development might our next research direction.

*CUC1* was detected in the whole area of the cotyledons. *35S::AtCUC1* transgenic plants showed small lobed cotyledons with defective vasculature (Hibara, Takada & Tasaka, 2003). The phenotype was similar to that of the *LcCUC2L* overexpressing plants. Both play role in cotyledon development. In the cotyledons of *35S::AtCUC1*, adventitious SAMs were observed on the adaxial surface of this region (Hibara, Takada & Tasaka, 2003). To date, we have not found that *LcCUC2L* could induce adventitious SAMs in transgenic *A. thaliana* plants. The above results show that the *LcCUC2L* and *CUC* genes seem to be homologous in sequence, but non-homologous in function. Moreover, differences in regulatory mechanisms exist between herbaceous and woody plants, and the expression of *LcCUC2L* in *L. chinense* may yield different results than that in *A. thaliana*. Therefore, to test the above hypothesis, it is necessary to transfer the *LcCUC2L* gene into *L. chinense*.

In summary, these observations suggest that *LcCUC2L* may play a critical role in leaf shape development in *L. chinense*. Our results lay a foundation for future studies of the mechanisms of leaf shape development and provide initial insight into the functions of the *CUC2* gene in *L. chinense*.

## Conclusions

In this study, histochemical GUS staining revealed that *LcCUC2L* was expressed in the cotyledons of *A. thaliana* seedlings, which indicated that *LcCUC2L* may play a role in cotyledon development. Ectopic expression of *LcCUC2L* resulted in long, narrow cotyledons without petioles and increased rosette leaf number. Further analysis showed that overexpression of *LcCUC2L* induced abnormal lamina epidermis cells and defective vascular tissue in cotyledons. Hormone determination and RT-qPCR results indicated that *LcCUC2L* affects leaf development by regulating the auxin content and the expression of genes related to auxin and leaf shape development. These findings indicate that *LcCUC2L* may influence leaf development in *L. chinense* and provide insights into the regulatory mechanisms of leaf development in *L. chinense*.

##  Supplemental Information

10.7717/peerj.12615/supp-1 Supplemental Information 1 The ORF sequences of *LcCUC2L*

10.7717/peerj.12615/supp-2 Supplemental Information 2 The promoter sequence of *LcCUC2L*

10.7717/peerj.12615/supp-3 Supplemental Information 3 The DNA detection of T1 transgenic lines and WT *A. thaliana.*

10.7717/peerj.12615/supp-4 Supplemental Information 4 All primers in this study

10.7717/peerj.12615/supp-5Supplemental Information 5Relative expression levels of *LcCUC2L* in leaves of different developmental stages in *L. chinense.*We applied the 2${}_{T}^{-\Delta \Delta C}$ method to analyze the data from relative quantification.

10.7717/peerj.12615/supp-6Supplemental Information 6The expression levels of *LcCUC2-like* under 200 µM IAA treatment in *L. chinense.*The expression levels of *LcCUC2-like* under 200 µM IAA treatment in *L. chinense.* We applied the 2${}_{T}^{-\Delta \Delta C}$ method to analyze the data from relative quantificationClick here for additional data file.

10.7717/peerj.12615/supp-7Supplemental Information 7The expression levels of *LcCUC2-like* in WT and in dependent transgenic linesWe applied the 2${}_{T}^{-\Delta \Delta C}$ method to analyze the data from relative quantification.

10.7717/peerj.12615/supp-8 Supplemental Information 8 The number of rosette leaves in WT and transgenic *A. thaliana* plants

10.7717/peerj.12615/supp-9 Supplemental Information 9 The IAA content of WT and transgenic *A. thaliana* plants

10.7717/peerj.12615/supp-10Supplemental Information 10The expression level of some genes in WT and transgenic *A. thaliana* plantsWe applied the 2${}_{T}^{-\Delta \Delta C}$ method to analyze the data from relative quantification.

## Additional Information and Declarations

### DNA Deposition

The following information was supplied regarding the deposition of DNA sequences: The sequences described here are available in the Supplemental Files and at GenBank: LcCUC2 MW629054.

### Data Availability

The following information was supplied regarding data availability: The raw data are available in the Supplementary Files.
