# Supplementary material for: Overexpression of the LcCUC2-like gene in Arabidopsis thaliana alters the cotyledon morphology and increases rosette leaf number
Source: PeerJ. 2022 Feb 2;10:e12615. doi: 10.7717/peerj.12615 (PMC8817629; doi:10.7717/peerj.12615)
Supplement: Supplemental Information 1 [file peerj-10-12615-s001.docx]

*>LcCUC2-like*

ATGGAAATCTTCAATCATTTCGACAGTGCTGAGACCCAACTTCCGCCGGGCTTCCGATTCCACCCAACTGACGAAGAGCTTATAACATACTATCTCATGAAGAAGGTCCTCGATAGCAGTTTCACAGGCCGAGCCATCGCTGAAGTGGACCTCAACAAGTGTGAGCCATGGCAGCTTCCCGAAAAGGCGAAGATGGGCGAAAAGGAATGGTATTTTTTCAGCCTTCGGGATCGAAAGTACCCAACTGGGCTTCGAACGAACAGAGCGACGGAAGCTGGGTATTGGAAAGCTACTGGAAAAGATAGAGAGATTTACAGTTCTAGAACTGGATCTCTGGTGGGGATGAAGAAGACCCTGGTATTTTACAAGGGGAGAGCTCCAAAGGGAGAAAAGAGCAACTGGGTCATGCATGAATACCGTCTTGAAGGGAAATTCTCTTATCATTACCTCACCAGAAGCTCTAAGGACGAGTGGGTCGTTTCCAGGGTTTTTCAGAAGAGCGGAGTTAATGGAGGGAAGAAGGCCCGTATCGGAGGCGGTGTTGGCTTCACCGACGTTGGATCTCCGTCGTCCTCATCGATTCCACCGCTGCTCGAATCATCACCGTACACAGCTGCGGCTGCGGCAGACAGGGAGAGCTGCTCCTACGAGAGCGAGAGCACAAGGGATCACGTGTCCTGCTTCTCCACCGTTGCCACAGCCTCGAACTTCAACCCTCAGACTATGGTTCCGGCTTCGATCCTCACTGACCTCACAGGGCAGTTTGGGAAGAACGGCGGAGGGGGGCCGGCGTTCCCAAGCCTGCGGTCGTTGCACGAGAATCTTCAGCTGCCATTCTTTTACTCAGCTGTGGCGCCCATGCCGACACTGGTGTCGGCGTCGGGTTCCGTGGGGCCCGGTGAGATGGGTGGGTGTAGTTTGGCTGGAAACTGGACCGGGGCTGATCAAAAGGTGGAGAGGAGCCGGCAGCAGCTGCAGATGGGAGGTACCGAGCTGGATTGCATGTGGACGTACTGA
