# Supplementary figures and images for: Overexpression of the LcCUC2-like gene in Arabidopsis thaliana alters the cotyledon morphology and increases rosette leaf number
Source: PeerJ. 2022 Feb 2;10:e12615. doi: 10.7717/peerj.12615 (PMC8817629; doi:10.7717/peerj.12615)

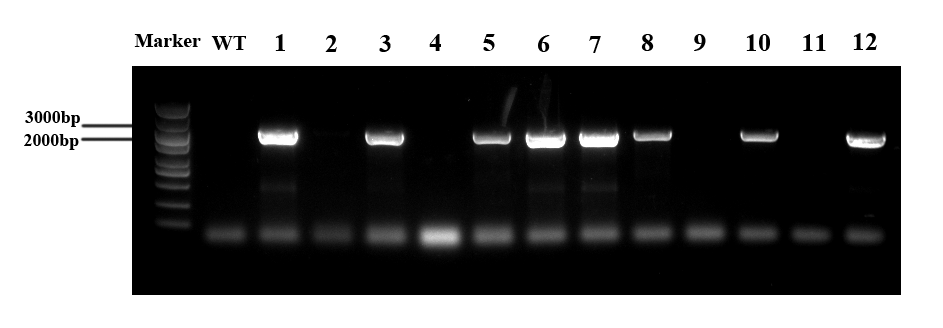

Supplement: Supplemental Information 3 [file peerj-10-12615-s003.tif]
