# Supplementary material for: Overexpression of the LcCUC2-like gene in Arabidopsis thaliana alters the cotyledon morphology and increases rosette leaf number
Source: PeerJ. 2022 Feb 2;10:e12615. doi: 10.7717/peerj.12615 (PMC8817629; doi:10.7717/peerj.12615)
Supplement: Supplemental Information 4 [file peerj-10-12615-s004.docx]

| **Primer name** | **Sequence (5′→3′)** | **Description** |
| --- | --- | --- |
| CUC2-F | CTTCCGATTCCACCCAACTGA | Middle fragment primer for *LcCUC2-like* |
| CUC2-R | GACCCACTCGTCCTTAGAGC | Middle fragment primer for *LcCUC2-like* |
| CUC2 3 race GSP1 | CGGGATCGAAAGTACCCAAC | 3′ RACE outer primer for *LcCUC2-like* |
| CUC2 3 race GSP2 | CCTCACCAGAAGCTCTAAGGACGA | 3′ RACE inner primer for *LcCUC2-like* |
| CUC2 5 race GSP1 | TTCCAGTAGCTTTCCAATACCCAGCTTCCGTCGCTCTGTTCGTTC | 5′ RACE outer primer for *LcCUC2-like* |
| CUC2 5 race GSP2 | CATCTTCGCCTTTTCGGGAAGC | 5′ RACE inner primer for *LcCUC2-like* |
| LcCUC2-F | ATGGAAATCTTCAATCATTTCGACA | Full length primer for *LcCUC2-like* |
| LcCUC2-R | TCAGTACGTCCACATGCAATCC | Full length primer for *LcCUC2-like* |
| CUC2-qF | CGATCCTCACTGACCTCACA | qRT-PCR for *LcCUC2-like* |
| CUC2-qR | CCAAACTACACCCACCCATC | qRT-PCR for *LcCUC2-like* |
| Actin97-F | TTCCCGTTCAGCAGTGGTCG | qRT-PCR for internal control |
| Actin97-R | TGGTCGCACAACTGGTATCG | qRT-PCR for internal control |
| LcCUC2-eGFPF | GAGAACACGGGGGACTCTAGAATGGAAATCTTCAATCATTTCGACA | GFP for *LcCUC2-like* |
| LcCUC2-eGFPR | GCCCTTGCTCACCATGGATCCGTACGTCCACATGCAATCCAGC | GFP for *LcCUC2-like* |
| LcCUC2-pBI121F | GGATCTTCCAGAGATTCTAGAATGGAAATCTTCAATCATTTCGACA | pBI121 for *LcCUC2-like* |
| LcCUC2-pBI121R | CTGCCGTTCGACGATGGATCCTCAGTACGTCCACATGCAATCC | pBI121 for *LcCUC2-like* |
| ProLcCUC2-F | AGCCGGTTCTTTTCTCATAGT | Promoter primer for *ProLcCUC2-like* |
| ProLcCUC2-R | TGGCTCACACTTGTTGAGGTC | Promoter primer for *ProLcCUC2-like* |
| ProLcCUC2-gusF | GGTACCCGGGGATCCACTTATGACGATGGATCATGC | 1301 for *ProLcCUC2-like* |
| ProLcCUC2-gusR | TTACCCTCAGATCTAGGAATCCCAAGCTGAAATCAA | 1301 for *ProLcCUC2-like* |
| LcCUC2-qF | ACCGCTGCTCGAATCATCACC | qRT-PCR for *LcCUC2-like* in *A. thaliana* |
| LcCUC2-qR | ATCGAAGCCGGAACCATAGTCT | qRT-PCR for *LcCUC2-like* in *A. thaliana* |
| AtYUC2-qF | ATGCCTTTTCCTTCAAGCTACCCT | qRT-PCR for *AtYUC2* |
| AtYUC2-qR  AtYUC4-qF  AtYUC4-qR | AACCCACACCGCCTATCGAAC  ACCTAAGACCGGACCAATTGAGC  AACTTTGCCCCGTTCCTCGTT | qRT-PCR for *AtYUC2*  qRT-PCR for *AtYUC4*  qRT-PCR for *AtYUC4* |
| AtYUC6-qF | AACTTTGCCCCGTTCCTCGTT | qRT-PCR for *AtYUC6* |
| AtYUC6-qR  AtAUX1-qF | TAGTCACGCGCCACATCCCA  AGCCATTGCCTCCATCATCCAC | qRT-PCR for *AtYUC6*  qRT-PCR for *AtAUX1* |
| AtAUX1-qR  AtPIN1-qF  AtPIN1-qR  AtPIN3-qF  AtPIN3-qR  AtPIN4-qF  AtPIN4-qR  AtKNAT6-qF  AtKNAT6-qR  AtKNAT2-qF  AtKNAT2-qR  DPA4-qF  DPA4-qR  Actin2-F | CTCAACAGTAACCGCGTGACC  CTCAAGGCTTATCTGCGACACC  ACCACCAGAAGCCATCATCGAG  AGCCGAAGCAAGTCAACGAA  CGACGAGAGCCCAAATAAGTCCA  GTCGCTATCTTCGCCGTCCCT  ATCATCCACTCCAAGCTACCGTT  TCCTCCGCCGGTGAAAATCGT  AACACGCTATCTCCGGTGGTGCT  CTACCGCTTTGTCCTCGGAGT  CGCTATTTCCATAGGCGCTCCC  CTCACAGCACACCGTCGTCT  GCAACAGAATCATCTCCGTCGTT  ACTCTCCCGCTATGTATGTCGCC | qRT-PCR for *AtAUX1*  qRT-PCR for *AtPIN1*  qRT-PCR for *AtPIN1*  qRT-PCR for *AtPIN3*  qRT-PCR for *AtPIN3*  qRT-PCR for *AtPIN4*  qRT-PCR for *AtPIN4*  qRT-PCR for *AtKNAT6*  qRT-PCR for *AtKNAT6*  qRT-PCR for *AtKNAT2*  qRT-PCR for *AtKNAT2*  qRT-PCR for *AtDPA4*  qRT-PCR for *AtDPA4*  qRT-PCR for *Actin2* |
| Actin2-R | ATTTCCCGCTCTGCTGTTGTGGT | qRT-PCR for *Actin2* |
| Ath-miR164a | TGGAGAAGCAGGGCACGTGCA | qRT-PCR for *miR164a* |
| AtCUC2-qF | AAAGGAAGAGCTCCGAAAGG | qRT-PCR for *AtCUC2* |
| AtCUC2-qR | CACAGTTGCTCCTCCTCCTC | qRT-PCR for *AtCUC2* |
